# Garcinol and Anacardic Acid, Natural Inhibitors of Histone Acetyltransferases, Inhibit Rhabdomyosarcoma Growth and Proliferation

**DOI:** 10.3390/molecules28145292

**Published:** 2023-07-08

**Authors:** Patrycja Tomasiak, Joanna Janisiak, Dorota Rogińska, Magdalena Perużyńska, Bogusław Machaliński, Maciej Tarnowski

**Affiliations:** 1 Institute of Physical Culture Sciences, University of Szczecin, 70-453 Szczecin, Poland; 2 Department of Physiology in Health Sciences, Pomeranian Medical University in Szczecin, 71-210 Szczecin, Poland; 3 Department of General Pathology, Pomeranian Medical University in Szczecin, 70-204 Szczecin, Poland; 4 Department of Experimental & Clinical Pharmacology, Pomeranian Medical University in Szczecin, 70-204 Szczecin, Poland

**Keywords:** epigenetics, RMS, histone acetyltransferases, inhibitors

## Abstract

Rhabdomyosarcoma (RMS) is a malignant tumour of the soft tissues. There are two main histopathological types: alveolar and embryonal. RMS occurs mainly in childhood and is a result of the deregulation of growth and differentiation of muscle cell precursors. There is an increasing amount of data indicating that numerous epigenetic alterations within chromatin and histone proteins are involved in the pathogenesis of this malignancy. Histone acetylation is one of the most important epigenetic modifications that is catalysed by enzymes from the group of histone acetyltransferases (HAT). In this study, the impact of the natural histone acetyltransferase inhibitors (HATi)—garcinol (GAR) and anacardic acid (AA)—on the biology of RMS cells was evaluated through a series of in vitro tests measuring proliferation, viability, clonogenicity, cell cycle and apoptosis. Moreover, using oligonucleotide microarrays and real-time PCR, we identified several genes whose expression changed after GAR and AA treatment. The examined HATi significantly reduce the invasive phenotype of RMS cells by inhibiting the growth rate, viability and clonogenic abilities. What is more, these substances cause cell cycle arrest in the G2/M phase, induce apoptosis and affect the genetic expression of the endoplasmic reticulum stress sensors. GAR and AA may serve as promising potential anti-cancer drugs since they sensitize the RMS cells to chemotherapeutic treatment.

## 1. Introduction

Rhabdomyosarcoma (RMS) belongs to the soft tissue sarcomas group and was first described by Weber in 1854, but formal classification was not made until 1946 [1]. It is commonly believed that this is a childhood disease because most cases occur in people under 18 (it ranks third among paediatric malignancies). RMS is most often diagnosed in children under the age of 5. A second, smaller peak of incidence is in adolescents aged 15–19 [2,3,4]. Microscopically, there are four main histological types of RMS: embryonal (ERMS); alveolar (ARMS); pleomorphic (PRMS); and spindle cell (ssRMS) [5,6]. The incidence risk increases with certain genetic syndromes, including Li-Fraumeni, Gardner, Warner and Noonan [7]. RMS arises because of numerous disturbances in the regulation of growth and differentiation of myogenic precursor cells, which may be a consequence of genetic and epigenetic modifications that affect cell differentiation, growth, proliferation and apoptosis [8,9]. Despite advanced chemotherapy combined with radiotherapy and surgery, patients with RMS have a poor prognosis caused by late diagnosis, metastasis and local recurrence [10].

Some substances of natural origin inhibit the development of malignant tumours through their effect on the epigenome. Restoring the epigenetic balance may be a chance to mitigate the malignant phenotype of cancer cells [11,12]. Natural products, such as garcinol and anacardic acid, seem to affect the enzymes controlling the process of histone acetylation and, more specifically, histone acetyltransferases (HATs) [13]. Garcinol (GAR) is a substance extracted from the dried peel of the *Garcinia indica* fruit that belongs to the polyisoprenylated benzophenones. It has been used in traditional medicine in East Asia and the Middle East. GAR has anticancer, antioxidant, anti-inflammatory, antiviral, antibacterial and antiparasitic properties. This substance is responsible for inhibiting HATs activity, primarily P300, CBP and PCAF enzymes. It may also affect signalling pathways involving NF-κB and STAT [14,15,16]. Anacardic acid (AA) is a bioactive phytochemical found in the shell of cashew nuts. It constitutes about 90% of CNSL (cashew nutshell liquid). The rest consists of cardanol and cardol. Considering its chemical structure, it is a mixture of organic compounds that are closely related to each other. Each of these compounds has salicylic acid with a saturated or unsaturated alkyl chain, containing from 15 to 17 carbon atoms in its structure. Antioxidant, antibacterial, antiparasitic and anticancer effects have been related to AA. Similar to GAR, AA has been found to be an effective inhibitor of HATs activity [13,17,18]. The RMS treatment strategy, mostly involving chemotherapy, is associated with many complications and significant side effects through high toxicity to healthy body cells. New ways of treating RMS are still greatly needed and expected. Our data indicate that AA and GAR increase the sensitivity of RMS cells to typically used chemotherapeutics and induce apoptosis through an endoplasmic reticulum (ER) stress-related mechanism.

## 2. Results

### 2.1. Anacardic Acid and Garcinol Reduce Viability and Proliferation of Rhabdomyosarcoma Cells

**Figure 1:**
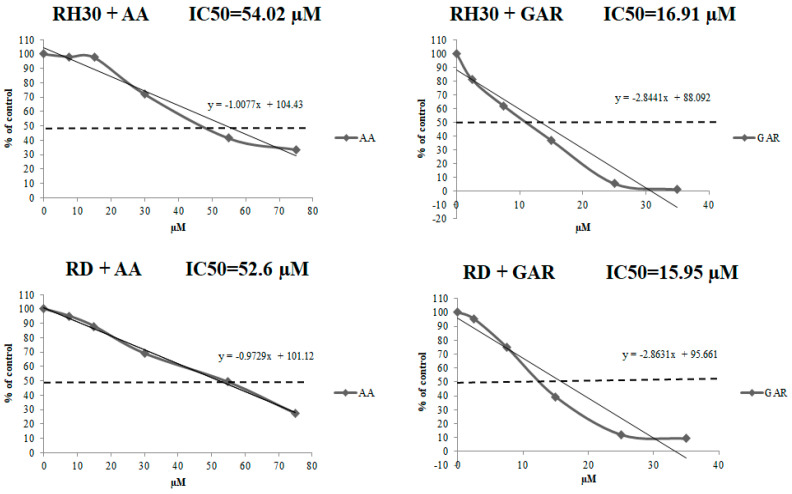
Evaluation of the viability of RH30 cells cultured in the presence of AA (7.5, 15, 30, 55, 75 µM) and GAR (2.5, 7.5, 15, 25, 35 µM). The viability index was calculated using the following formula: (cell viability (% of control) = [(A tested-A blank/A control-A blank)] × 100%). The IC50 value was calculated from the trend line equation. A representative result from three independent experiments is shown.

**Figure 2:**
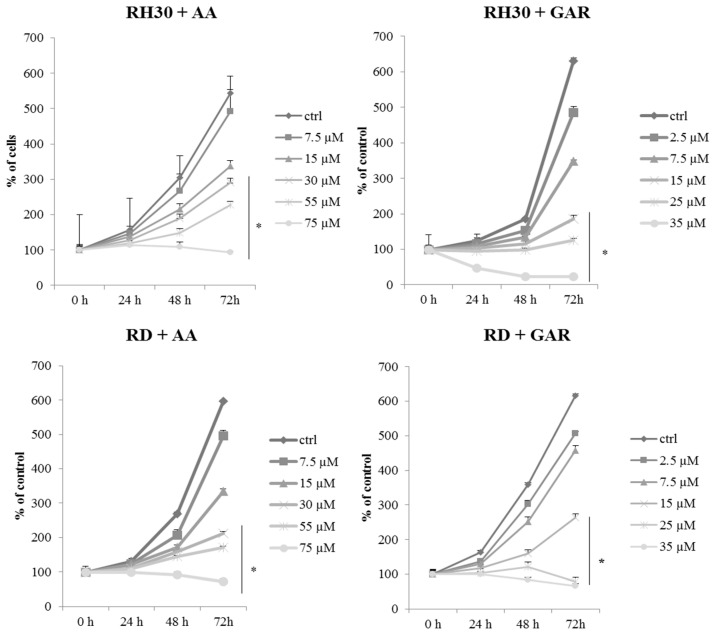
Assessment of the proliferative potential of rhabdomyosarcoma cells in the presence of a concentration gradient of anacardic acid (7.5, 15, 30, 55, 75 µM) and garcinol (2.5, 7.5, 15, 25, 35 µM). The analysis was performed at times: 0; 24; 48; 72 h. The proliferation rate index was calculated in relation to control cells (RPMI-1640 or DMEM medium with 10% FBS). The result of three independent experiments is presented. (*) = *p* < 0.05 as compared with control (untreated) cells.

To evaluate the effect of GAR and AA on RMS viability, we applied WST-1 over a broad range of tested compound concentrations. RH30 and RD cells were cultured in a medium with AA (7.5, 15, 30, 55, 75 µM) or GAR (2.5, 7.5, 15, 25, 35 µM). In both cases, a similar tendency was observed, namely, the percentage of viable alveolar (ARMS) and embryonal (ERMS) cells decreased with increasing concentrations of AA and GAR (Figure 1). We calculated the IC50 index: for AA and RH30 cells, the IC50 was 54.02 µM, and for RD cells, the IC50 was 52.6 µM, while for GAR, it was 16.91 µM and 15.95 µM, respectively. Then, the effect of AA and GAR on the proliferation of RH30 and RD cells was assessed (Figure 2), and a decrease in the growth kinetics of the examined cells was noted in just 24 h after the addition of the compounds. The decrease in the proliferation rate of RH30 and RD cells was proportional to the increase in the HAT inhibitors (HATi) dose. Statistically significant differences in the proliferation rate of the tested cells in relation to the control cells were observed at doses of 30, 55 and 75 µM for AA and 15, 25 and 35 µM for GAR.

### 2.2. Anacardic Acid and Garcinol Reduce Clonogenicity of Rhabdomyosarcoma Cells

**Figure 3:**
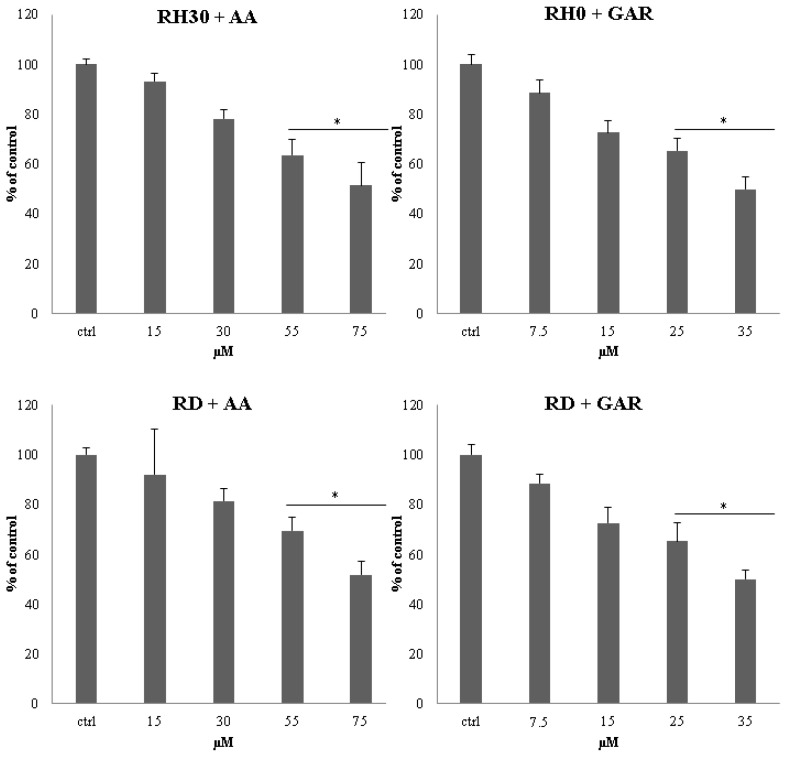
Assessment of clonogenicity of RMS cells in the presence of AA: 15; 30; 55; 75 µM and GAR: 7.5; 15; 25; 35 µM. Colonies were counted by light microscopy 3 weeks after the start of the experiment. The clonogenic index is presented as the ratio of cells incubated in the presence of the test substances to control cells. A representative result from 3 experiments is shown. (*) = *p* < 0.05, as compared to the number of control (untreated) cells.

In the subsequent in vitro test, we evaluated the effect of tested compounds on anchorage-independent proliferation. We noted a significant reduction in the number of colonies formed in the presence of AA and GAR in the tested cell lines as compared to the control (untreated cells). The trend was proportional to the increase in the concentration of the compound used (Figure 3 and Figure S1). Statistically significant changes for the RH30 and RD cells were detected in the two highest concentrations of both AA and GAR. AA at a concentration of 55 µM reduced the clonality of RH30 cells to 64% and RD to 69%. At the highest concentration of AA, a reduction in clonality of up to 52% was observed for both cell lines. A similar situation was observed in the case of GAR. A concentration of 25 µM reduced the clonality of RH30 cells to 65% and RD to 62%. GAR used in the highest concentration reduced colony-forming capacity to 50% in ARMS cells and 47% in ERMS.

### 2.3. Anacardic Acid and Garcinol Affect the Cell Cycle and Induce Apoptosis of RH30 and RD Cells

**Figure 4:**
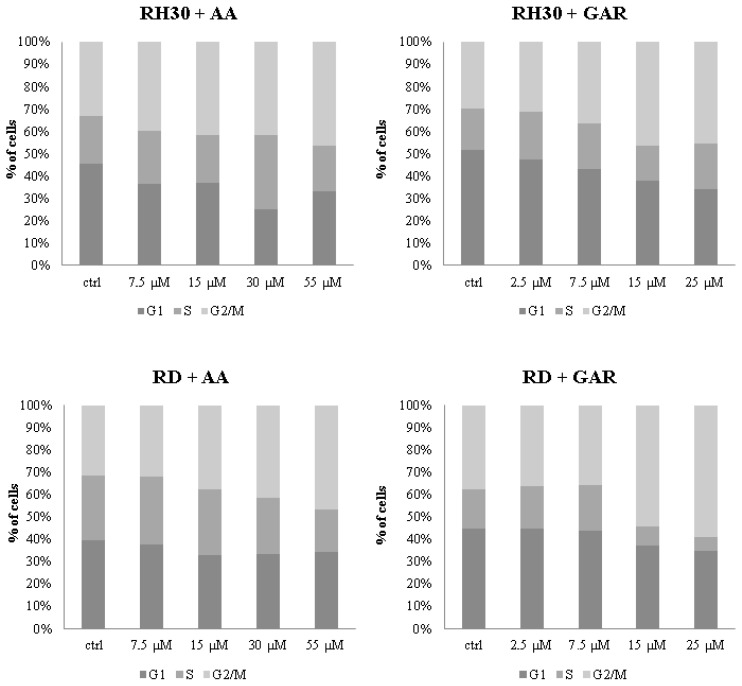
Evaluation of the cell cycle of RMS cells under the influence of increasing concentrations of AA: 7.5; 15; 30; 55 µM and GAR: 2.5; 7.5; 15; 25; 35 µM. Control cells were cultured in a medium supplemented with 10% bovine serum. The result of 3 independent experiments is presented.

The decrease in the multiplication rate of RMS cells and the reduction in colony numbers under the influence of AA and GAR may be related to their effect on the cell cycle (Figure 4). In the case of RH30 cells incubated with AA, statistically significant differences in the cell cycle shift were observed at a concentration of 55 µM, where the percentage of cells in the G2/M phase increased to 46.35% (the percentage of control cells oscillated around 33%) (Figure 4). This change was accompanied by a decrease in the number of cells that were in the G1 phase (from 45.24% for control to 33.31% for 55 µM AA). Slight fluctuations were observed in the S phase. The same trend was observed in the RD cell line, where the highest concentration of AA caused the cells to arrest in the G2/M phase of the cell cycle (31% control, 43% 55 µM AA) (Figure 4 and S2). The G1 phase did not change significantly, while the S phase was reduced from 29.18% for the control to 17.27% for 55 µM AA. GAR had a stronger effect on the cell cycle (Figure 4). In the RH30 line, a significant reduction in the number of cells in the G1 phase was observed at concentrations of 15 and 25 µM (control 51.63%, 15 µM GAR 34.17%, 25 µM GAR 34.12%). In addition, an increase in the percentage of cells remaining in the G2/M phase was observed (control 29.69%, 15 µM GAR 41.96%, 25 µM GAR 45.37%). In the RD cell line, there was a decrease in the number of cells in the S phase (control 17.56%, 15 µM GAR 8.68%, 25 µM GAR 6.12%) and an increase in the number of cells in the G2/M phase (control 37.49%, 15 µM GAR 54.42%, 25 µM GAR 59.04%). The above changes reached statistical significance. In addition, slight fluctuations were observed in the G1 phase for the RD line and the S phase for the RH30 line (Figure 4).

**Figure 5:**
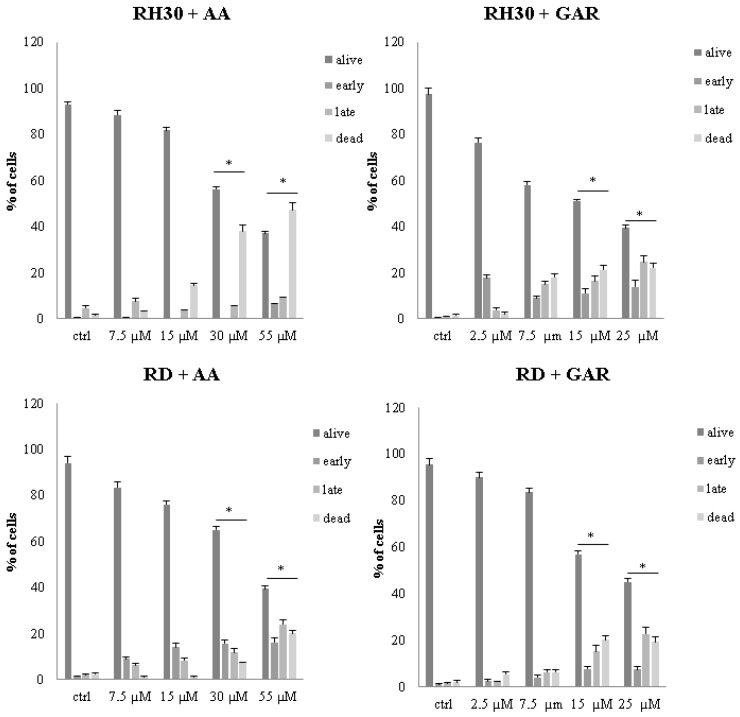
Assessment of apoptosis of RH30 and RD cells in the concentration gradient of AA (7.5, 15, 30, 55 µM) and GAR (2.5, 7.5, 15, 25 µM). The result from 3 independent experiments is presented. (*) = *p* < 0.05, as compared to the number of control (untreated) cells.

GAR and AA application led to a significant induction of apoptosis in RMS cells. Doses of 30 and 55 µM for AA and 15 and 25 µM for GAR were the most effective in triggering an apoptotic process (Figure 5 and Figure S3).

### 2.4. Anacardic Acid and Garcinol Induce Changes in the Transcriptome of RH30 and RD Cells

**Figure 6:**
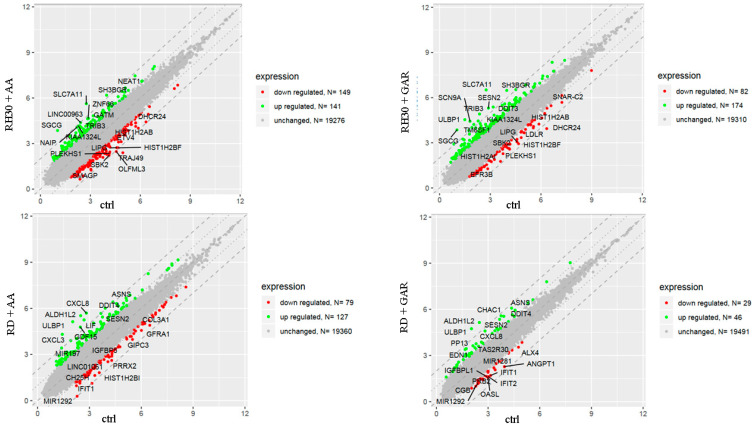
Graphical representation of the analysis of expression of altered genes in RH30 and RD cells incubated with AA and GAR compared to control cells. Each gene is marked with a dot. Genes marked in green increased their expression at least 2-fold, while genes with at least a 2-fold decrease in expression compared to controls are represented by red dots.

The oligonucleotide arrays analysis allowed concluding that GAR and AA have a significant impact on multiple cellular processes. The genes most strongly regulated by AA and GAR are shown in Figure 6. 

**Figure 7:**
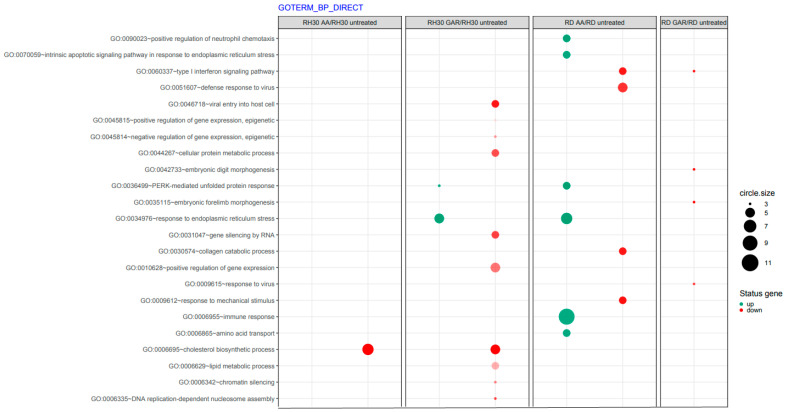
Inventory of biological processes affected by garcinol (GAR) and anacardic acid (AA) in ARMS and ERMS cell lines based on gene ontology (GO). The size of each dot represents the number of genes involved; fold difference >2 or <−2; *p* < 0.05, FDR-adjusted *p*-value was less than 0.2.

It was noticed that these compounds have a strong effect on the signalling pathways involved with oxidative stress (GO:0034976, GO:0036499, GO:070059) and stimulate the immune response (GO:0006955) (Figure 7). Inducing a response to ER stress can occur through the PERK (RNA-dependent protein kinase (PKR)-like ER kinase) signalling pathway and then direct cancer cells into apoptosis. Since cancer cells bypass the programmed death pathway, substances that induce apoptosis become attractive candidates for antineoplastic drugs.

**Figure 8:**
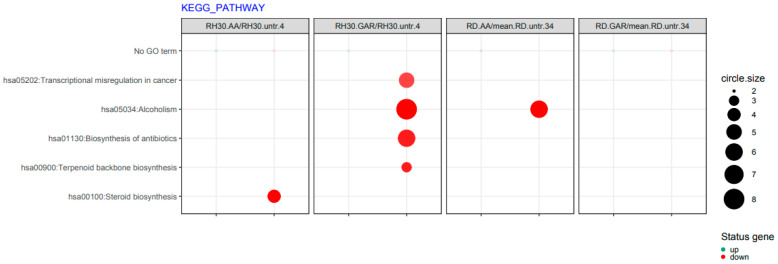
List of functions of biological processes altered by GAR and AA in RMS cells, obtained from the KEGG collection. The size of the circle is directly proportional to the number of genes involved in the process. The size of each dot represents the number of genes involved; fold difference >2 or <−2; *p* < 0.05, FDR-adjusted *p*-value was less than 0.2.

In addition, it was observed that after the use of AA and GAR, the transcriptional misregulation characteristic of carcinogenesis is reduced (hsa05202) (Figure 8).

**Figure 9:**
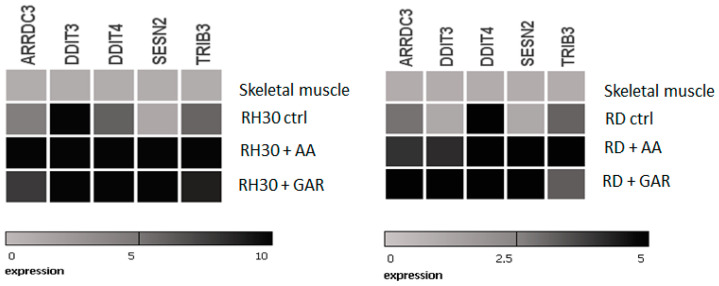
Heat maps of RQ-PCR results showing expression of ER-stress-related genes selected by microarray analysis in RMS cells treated with AA and GAR. The figure shows the activity of *ARRDC3*, *DDIT4*, *SESN2* and *TRIB3* genes in striated muscle, RH30 cell line and RH30 cells cultured in the presence of AA (55 µM) or GAR (15 µM).

Based on lists of genes that have most changed their expression in RMS cells under the influence of AA and GAR, these five were selected: *ARRDC3* (arrestin domain containing 3); *DDIT3* (DNA damage-inducible transcript 3), *DDIT4* (DNA damage-inducible transcript 4); *SESN2* (sestrin 2); and *TRIB3* (tribbles pseudokinase 3). All five genes had lower expression in RMS cells than normal muscle tissue. The products of these genes seem to be involved in the ER-stress mechanisms. Subsequently, we checked their expression upon exposure to GAR and AA (Figure 9) by RQ-PCR. It was noticed that the use of the tested HATi caused the reactivation of genetic expression. This experiment confirmed the results from the microarray analysis. In addition, it was shown that the use of AA and GAR caused a change in the expression profile of these genes towards the expression occurring in the control tissue of normal muscle.

### 2.5. Anacardic Acid and Garcinol Sensitize RMS Cells to Chemotherapeutic Agents

**Figure 10:**
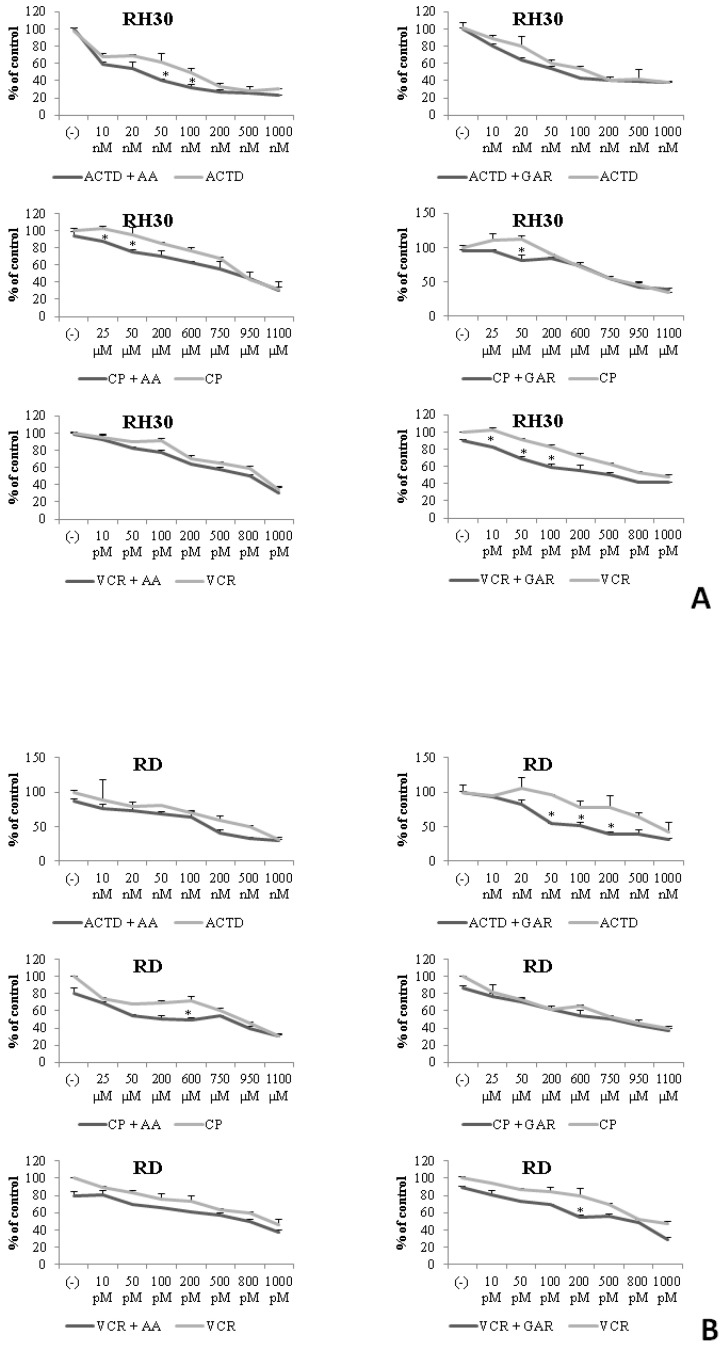
Concomitant treatment of RMS cells with chemotherapeutics (actinomycin D (ACTD), cyclophosphamide (CP), vincristine (VCR) and subtoxic concentration of AA (55 µM) or GAR (15 µM). Panel (**A**) shows the proliferative potential of RH30 cells after co-treatment with selected doses of chemotherapeutic agents and AA or GAR. Panel (**B**) presents the effect of simultaneous stimulation of RD cells with selected doses of chemotherapeutics and AA or GAR on their proliferation rate. (ACTD—actinomycin D, CP—cyclophosphamide, VCR—vincristine). All experiments were repeated three times with similar results. (*) = *p* < 0.05 compared to cells subjected to the chemotherapeutic treatment only.

A commonly used treatment for cancer is chemotherapy. In our study, we used three antineoplastic drugs: actinomycin D; cyclophosphamide; and vincristine. We checked the effect of the simultaneous use of the chemotherapeutics and natural inhibitors of HATs (AA and GAR) on the proliferation of RMS cells. RH30 and RD cells were treated with increasing doses of actinomycin D, cyclophosphamide and vincristine and subtoxic doses of AA (55 µM) or GAR (15 µM) for 72 h. We found that AA and GAR sensitized RH30 cells (Figure 10A) and RD cells (Figure 10B) to all three chemotherapeutic agents.

## 3. Discussion

There is increasing evidence that epigenetic changes are involved in the pathogenesis of RMS. Epigenetic dysregulation occurs at the level of methylation, acetylation, deacetylation and miRNA [19]. Epigenetic changes control many biological processes, from embryogenesis to determination of cell fate. The reversibility of epigenetic modifications makes them an attractive target in modern anticancer therapies [20]. The epigenetic drugs most frequently described in the literature are inhibitors of DNA methyltransferases: azacitidine [21] and decitabine [22], and inhibitors of histone deacetylases: trichostatin A [23]; and SAHA [22]; and protein methyltransferase inhibitors: AMI1 and SAH [24]. In recent years, there has been limited advancement in identifying new therapeutic agents and approaches to treating RMS [25]. The standard treatment for RMS for more than 40 years has been multi-agent chemotherapy, mostly the combined use of vincristine, dactinomycin and cyclophosphamide (VAC). Other implemented treatment systems like CEV, IVA or IVE have not increased overall survival but were associated with significant side effects [26]. Hence, there is a continuous need for the exploration of new substances and compounds with higher efficacy and lower risks of side effects. This study aimed to investigate the influence of natural inhibitors of histone acetyltransferases, GAR and AA, on the biology of RMS cells. 

To date, it has been shown that GAR and AA inhibit the growth of cancer cells, including breast, pancreas and prostate [27,28,29,30,31]. Our results showed that AA and GAR reduced the rate of multiplication of RMS cells (Figure 1 and Figure 2). Proliferation, viability and clonogenicity tests showed that GAR and AA significantly reduced the RMS growth potential (Figure 3 and Figure 4). This effect was dependent on the dose of the tested compounds. The IC50 determined in the viability test for GAR was about 15 µM and AA about 54 µM, demonstrating that garcinol is more effective at lower doses (Figure 1). This result is similar to the results obtained by other research groups [32,33,34,35,36,37,38]. In addition, we tested whether AA and GAR sensitize RMS cells to chemotherapeutic agents commonly used in RMS treatment (i.e., VAC) (Figure 10). These results correspond with other reports regarding the increased effectiveness of anticancer treatment (i.e., radio- and chemotherapy) in the presence of AA and GAR [36,39].

AA and GAR effectively inhibit cell division and halt cells in different cell cycle phases. The cell cycle is regulated by a system of checkpoints whose task is to condition cell transition to the next phase of the cycle, as well as to monitor the course of processes occurring during individual phases. The regulation of the cell cycle takes place through the interplay of excitatory and inhibitory factors. Cyclins and cyclin-dependent kinases form complexes that regulate the entry of cells into subsequent phases of the cell cycle. Cancer cells are characterized by changes in DNA. Disruption of the repair mechanisms results in the accumulation of errors that are duplicated, thus conditioning the malignant phenotype of the cells. Cells, defending themselves against changes occurring within the DNA, can block the division cycle. However, in the case of cancer cells, this mechanism does not work properly. The phenomenon of blocking the cell cycle is used in oncological treatment, and the targets of anti-cancer drugs are the cell cycle checkpoints. Stopping cell division may prevent the reproduction of erroneous genetic information contained in cancer cells [40]. In our studies, RMS cells were arrested in the G2/M phase of the cell cycle after using the tested HATi (Figure 4). Researchers that previously tested the effects of GAR and AA on the cell cycle noted the arrest in the G1, S and G2/M phases [33,38,41,42]. It is likely that different cell types respond differently to particular HATi. 

Apoptosis is a genetically controlled process. Its purpose is to remove cells whose genetic material has been irreversibly damaged. Such cells may be the source of neoplastic transformation. Abnormal cells show changes in gene expression profiles that encode pro- and anti-apoptotic factors. The peculiarity of these cells is that they develop resistance to apoptosis. Our experiments showed that natural HAT inhibitors—AA and GAR—have a strong pro-apoptotic effect on RMS cells (Figure 5). This effect has also been confirmed in other types of neoplasms, including breast and prostate cancer [38,43,44]. It has been proven that the process of apoptosis can be triggered by the activation of ER stress proteins. The tumour microenvironment, which is characterized by hypoxia and energy deficiency, may disrupt the role of the ER in maintaining cellular homeostasis. Activation of ER stress occurs because of the increasing amount of misfolded or unfolded proteins in the ER. This process leads to the activation of several stress receptors, such as PERK (RNA-dependent protein kinase (PKR)-like ER kinase), ATF6 (activating transcription factor 6) and IRE1 (inositol requiring enzyme 1), which are responsible for the activation of the adaptive stress response pathway (UPR, unfolded protein response). This mechanism is designed to restore cellular homeostasis. In a situation where the ER stress is prolonged, the apoptotic pathway is activated. It is assumed that the promotion of apoptosis resulting from ER stress may serve as a promising anticancer strategy [29]. Our data indicated that GAR and AA increased the expression of genes promoting programmed cell death through the mechanism of ER stress (Figure 6, Figure 7 and Figure 8). The increased expression of DDIT3, DDIT4, TRIB3 and SESN2 genes in treated RMS cells was confirmed by both oligonucleotide microarray analysis and RQ-PCR (Figure 9). The protein products of these genes are involved in the regulation of apoptosis resulting from prolonged ER stress. Our results correspond with the results obtained by Tan et al., who proved that AA induces apoptosis of prostate cancer cells by affecting the induction of, among others, factors causing ER stress (i.e., BiP, DDIT3, p-eIF2α) and inhibiting the p-Akt and mTOR [29]. Therefore, the pro-apoptotic effect of HATi may be related to an increased expression of genes involved in the regulation of ER stress.

## 4. Materials and Methods

### 4.1. Cell Lines

The material used for the study was two established human RMS cell lines: the ARMS type model (RH30) and the ERMS type model (RD). The cell lines were purchased from the American Type Culture Collection. Cells were cultured according to manufacturer instructions in RPMI-1640 medium (RH30) and in DMEM medium (RD) (both Sigma-Aldrich, USA). The media was supplemented with streptomycin (10 µg/mL), penicillin (100 IU/mL) and 10% Foetal bovine serum (FBS) purchased from Gibco. Cells were stored in an incubator at 37 °C, humidity 95% and CO_2_ 5%. Cell medium was replaced every 48 h.

### 4.2. Viability Test

The viability of RMS cells in the presence of GAR and AA was tested using the WST-1 test. The essence of the test is the colorimetric determination of the number of living cells in the entire population. RH30 and RD cells were seeded in triplicate on a flat-bottomed 96-well plate at 2.5 × 10^3^. Control cells were suspended in a medium with 10% FBS, and test cells were cultured in the presence of GAR (2.5, 7.5, 15, 25, 35 µM) (Tocris) and AA (7.5, 15, 30, 55, 75 µM) (Sigma-Aldrich) for 72 h (the medium was changed every 24 h). After this time, 10 μL of WST-1 reagent was added to each of the samples, followed by a 30-min incubation at 37 °C and shaking for 1 min. The principle of the operation of the reaction is to convert the red tetrazolium salt to yellow formazan under the influence of metabolically active cells. The amount of formazan generated was determined using a spectrophotometer on a microplate reader (Infinite 200 Pro, Tecan, Switzerland) at 450 and 650 nm. The WST-1 test allowed determining the IC50 value of the tested substances. Cell viability was calculated using the formula: Cell viability (% of control) = [(Atest − Ablank/Acontrol − Ablank)] × 100%.

### 4.3. Proliferation Test and Chemotherapy Treatment

To test the proliferation rate of RH30 and RD cells, they were plated at 7 × 10^4^ per well in a flat-bottomed 24-well plate and cultured in the appropriate medium: cells supplemented with 10% FBS were controls, and GAR (2.5, 7,5, 15, 25, 35 µM) or AA (7.5, 15, 30, 55, 75 µM). The culture medium was changed every 24 h. The influence of HATi on the rate of growth kinetics of RMS cells was tested at 0, 24, 48 and 72 h from the start of culture. At designated time points, cells were trypsinized and counted using a Navios flow cytometer (Beckman Coulter, Brea, CA, USA). The analysis for each sample lasted 60 s. Two replicates were performed for one sample in three independent experiments.

The proliferation test was also performed with or without GAR (15 µM) and AA (55 µM) and selected chemotherapeutics, i.e., vincristine (10 pM, 50 pM, 100 pM, 200 pM, 500 pM, 800 pM, 1000 pM), actinomycin D (10 nM, 20 nM, 50 nM, 100 nM, 200 nM, 500 nM, 1000 nM), and cyclophosphamide (25 µM, 50 µM, 200 µM, 600 µM, 750 µM, 950 µM, 1100 µM). All chemotherapeutics were purchased from Sigma-Aldrich. The cell number was counted at 24, 48 and 72 h after culture start, using a cytometer.

### 4.4. Clonogenicity Test

The colony-forming ability of RH30 and RD cells was tested in the presence of GAR and AA. The experiment was carried out using agar. Twenty-four-well plates were covered with 0.5% agar mixed with the appropriate growth medium. The counted cells were then plated at 1.25 × 10^3^ per well in combination with 0.35% agar and either DMEM or RPMI-1640. Every 3 days, standard medium was added to the cells at 250 μL/well along with GAR (2.5, 7.5, 15, 30 μM) or AA (7.5, 15, 3, 75 μM). The control cells received medium containing 10% FBS. Two replicates of each sample were performed. After 3 weeks, unstained colonies were counted using an optical microscope (Zeiss, Baden-Württemberg, Germany). The experiment was repeated three times. 

### 4.5. Cell Cycle Analysis

Vybrant^®^ DyeCycle™ Orange Stain (ThermoFisher Scientific, Waltham, MA, USA) was used to assess the cell cycle, which can be used to divide the cell population into three main phases of the cell cycle: G0/G1; S; and G2/M. Cells were seeded in a 6-well plate at 5 × 10^5^ and cultured in standard medium or various concentrations of GAR (2.5, 7.5, 15, 25 μM) and AA (7.5, 15, 30, 55 μM). After 48 h of incubation with the test substances, the cells were trypsinized, centrifuged (1200 rpm/RT/8′), suspended in PBS (EURx) and stained with Vybrant^®^ DyeCycle™. Analysis was performed using a Navios flow cytometer (Beckman Coulter, Brea, CA, USA). The results are presented as the percentage of responses relative to controls. Results were compiled using ModFit LT 4.1 software and presented as the percentage of control wells. Two replicates were performed for each sample in three independent experiments.

### 4.6. Apoptosis

RMS cell apoptosis was detected using annexin V and propidium iodide. Cells were seeded in a 6-well flat-bottom plate at 0.5 × 10^6^ per well. After 24 h, preselected concentrations of GAR (2.5, 7.5, 15, 25 μM) and AA (7.5, 15, 30, 55 μM) were added to the cells. Cells were stained after 48 h with annexin V-FITC (5 μL) and PI (5 μL) in 1× binding buffer (10 mM HEPES, pH 7.4, 140 mM NaOH, 2.5 mM CaCl_2_). After 15 min incubation at room temperature in the dark, cytometric analysis (Navios, Beckman Culture) was performed. Early apoptotic cells were those that stained with annexin V, while populations in late apoptosis or necrosis stained with both annexin V and propidium iodide. The results were analysed using the Kaluza 2.2.1 software and presented as a percentage relative to the control. The experiment was performed in three repetitions.

### 4.7. Oligonucleotide Microarrays

The material for the microarray analysis was RNA isolated from RH30 and RD cells cultured in the presence of selected concentrations of GAR (15 µM) and AA (55 µM). Control cells were cultured in standard medium supplemented with 10% bovine serum. To minimize individual differences between samples, pools of material from three isolated RNAs of each group were made. Reverse transcription and DNA amplification were performed using the GeneChip™ WT PLUS Reagent Kit (ThermoFisher Scientific). Single-stranded DNA was then fragmented and labelled using the GeneChip™ WT Terminal Labeling Kit (Affymetrix, ThermoFisher Scientific). Reverse transcription and DNA amplification were performed using the GeneChip™ WT PLUS Reagent Kit (ThermoFisher Scientific). Single-stranded DNA was then fragmented and labelled using the GeneChip™ WT Terminal Labeling Kit (Affymetrix, ThermoFisher Scientific). In the next step, GeneChip™ Hybridization, Wash, and Stain Kit and GeneChip™ Human Gene 2.1 ST Array Strip (ThermoFisher Scientific) were used for the hybridisation of the coding DNA strand with the oligonucleotide matrix. Data reading was performed using the GeneAtlas™ System platform, and measurement of the fluorescence intensity of phycoerythrin was performed using a visualisation station (Affymetrix Gene Atlas ™ Imaging Station). Finally, a CEL catalogue was created, which was the output file for further bioinformatics analysis. CEL files were imported into the Bioconductor application. Background correction, normalisation and summary of the raw files were performed using the RMA algorithm. In the next step, the results were combined with a descriptive library, resulting in a complete list of genes. The selection of results between the tested samples was conducted after at least a two-fold change in gene expression in relation to the control samples.

### 4.8. Real-Time Quantitative Reverse Transcription PCR (RQ-PCR)

Total RNA was isolated from cells treated with AA or GAR and from cell controls with the RNeasy Kit (Qiagen, Germany). The real-time PCR reaction was carried out on the ABI 7500 Fast device (Applied Biosystems, Waltham, MA, USA) with Power SYBR Green PCR Master Mix Reagent (ThermoFisher Scientific) under the following conditions: 95 °C (15 s); 40 cycles at 95 °C (15 s); 60 °C (1 min). The comparative method ΔΔCt allowed for determining the relative expression of the studied gene (R). It was necessary to apply normalisation to β-2 microglobulin (reference gene) and calibrator. The expression of the following genes was analysed: ARRDC3; DDIT3; DDIT4; SESN2; and TRIB3. Primer sequences are available upon request.

### 4.9. Statistical Analysis

The results are presented as the mean ± standard error of the mean (SEM). Statistical data analysis was performed using the non-parametric Mann-Whitney or Student’s *t*-test, with *p* < 0.05 considered significant.

## 5. Conclusions

Our study provides evidence that AA and GAR—natural inhibitors of histone acetyltransferases significantly reduce the malignant behaviour of RMS cells by reducing the growth rate, viability and clonogenicity, as well as inducing apoptosis. Supposedly AA and GAR act through the ER stress-induction sensors. Moreover, these compounds increase the sensitivity of RMS cells to chemotherapeutics, which may be promising in future anti-cancer treatment applications.

## Data Availability

The data presented in this study are available on request from the corresponding author.

## Supplementary Materials

The following supporting information can be downloaded at: https://www.mdpi.com/article/10.3390/molecules28145292/s1, Figure S1: Assessment of clonogenicity of RMS cells in the presence of AA: 15, 30, 55, 75 μM and GAR: 2.5; 7.5; 15; 25; 35 μM. Clonogenicty assay results plotted against number of clones. (*) = *p* < 0.05 vs control (untreated) cells; Figure S2. Representative graphs of the evaluation of the cell cycle shift of RMS cells under the influence of high doses of AA (55 μM) and GAR (25 μM). Percentages indicate number of cells in particular phase of the cell cycle. Figure S3. Representative plots of the evaluation of the apoptosis of RMS cells under the influence of high doses of AA (55 μM) and GAR (25 μM). Percentages indicate number of cells in particular stages of apoptosis.
