# Supplementary material for: Garcinol and Anacardic Acid, Natural Inhibitors of Histone Acetyltransferases, Inhibit Rhabdomyosarcoma Growth and Proliferation
Source: Molecules. 2023 Jul 8;28(14):5292. doi: 10.3390/molecules28145292 (PMC10383693; doi:10.3390/molecules28145292)
Supplement: Supplementary file 1 [file molecules-28-05292-s001.zip › molecules-2396306-supplementary.pdf]

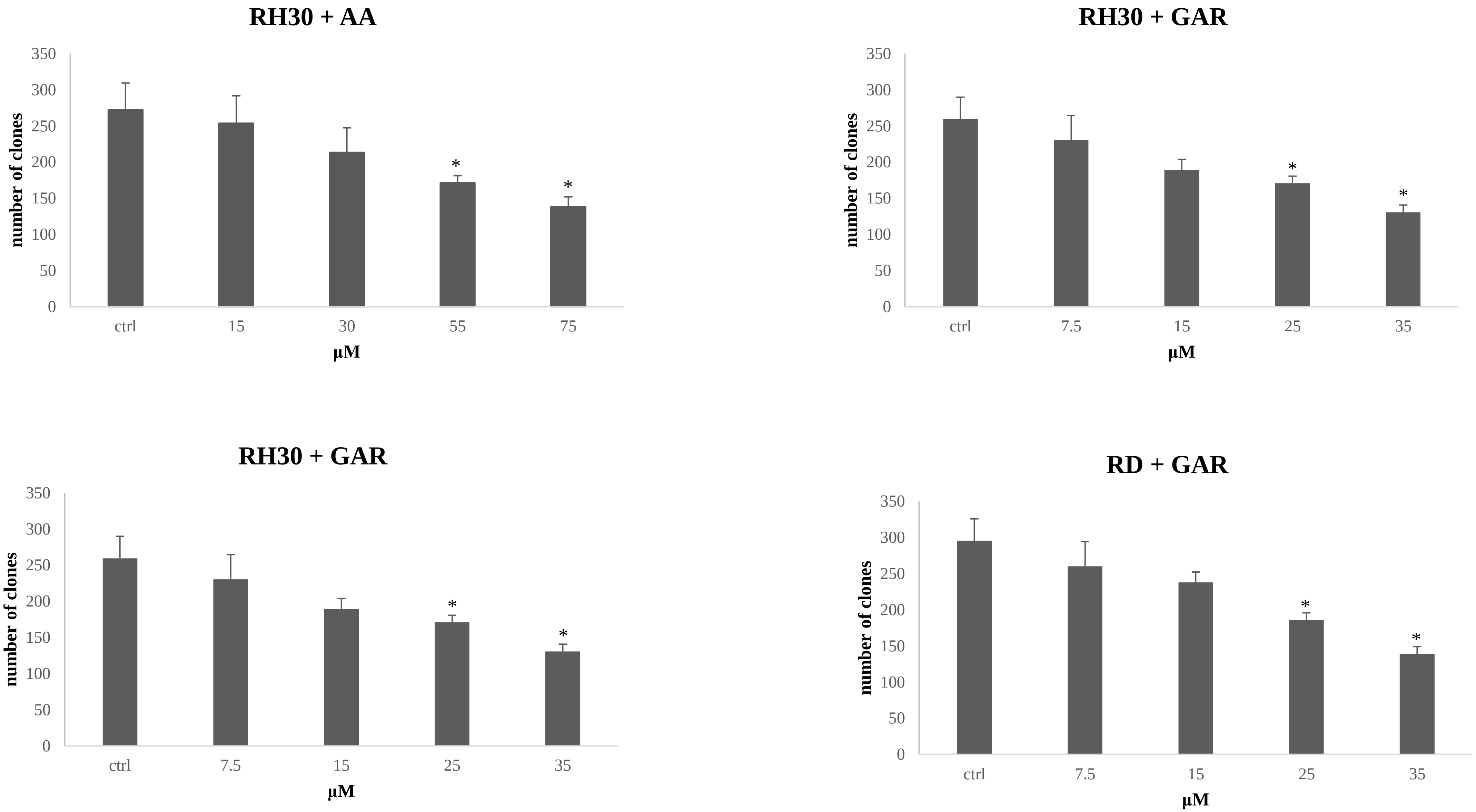

Supplementary Figure S1. Assessment of clonogenicity of RMS cells in the presence of AA: 15, 30, 55, 75 µM and GAR: 2.5; 7.5; 15; 25; 35 µM. Clonogenicty assay results plotted against number of clones. (*) = $p<0.05$ vs control (untreated) cells.

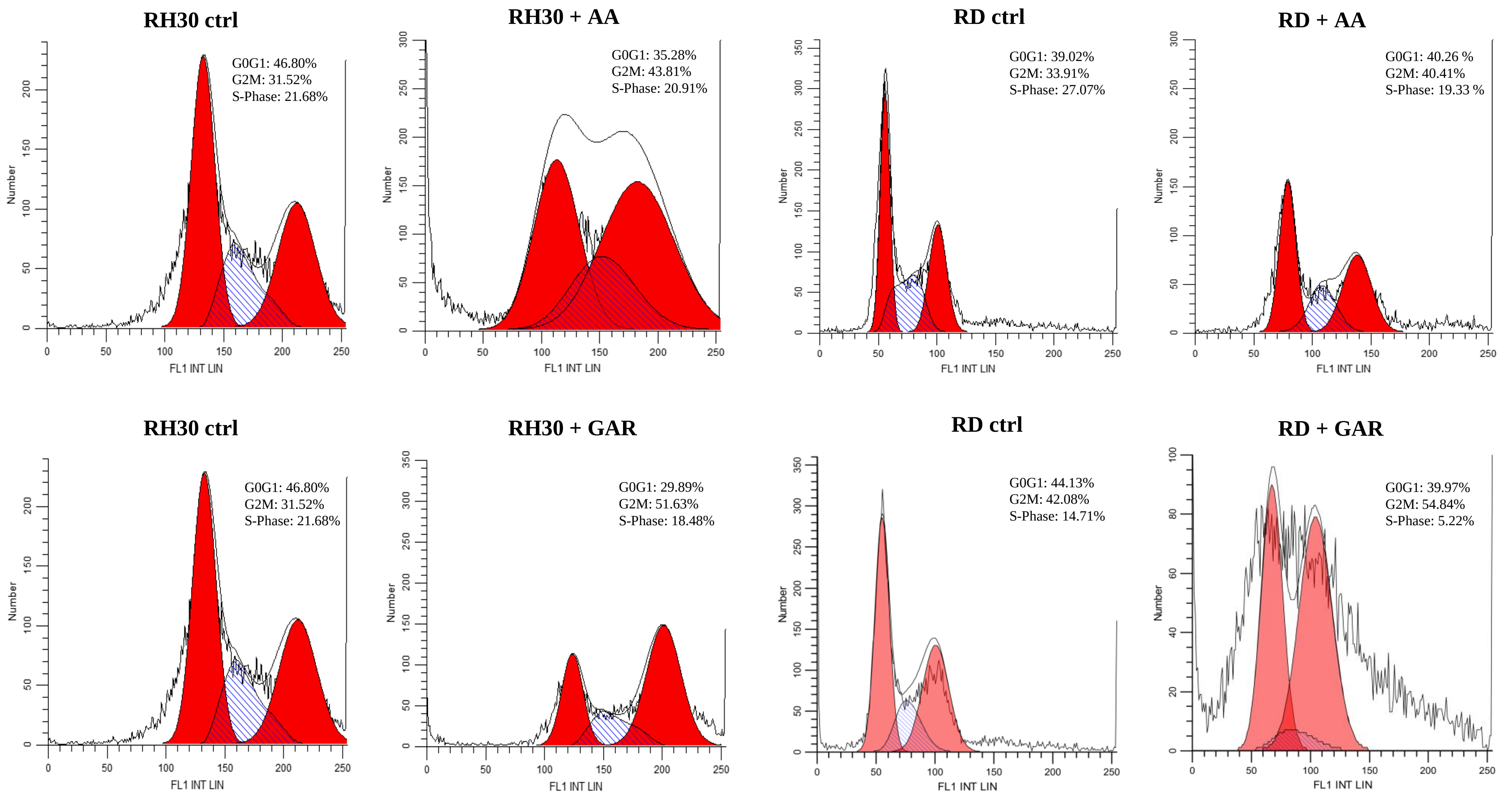

Supplementary Figure S2. Representative graphs of the evaluation of the cell cycle shift of RMS cells under the influence of high doses of AA (55 µM) and GAR (25 µM). Percentages indicate numer of cells in particular phase of the cell cycle.

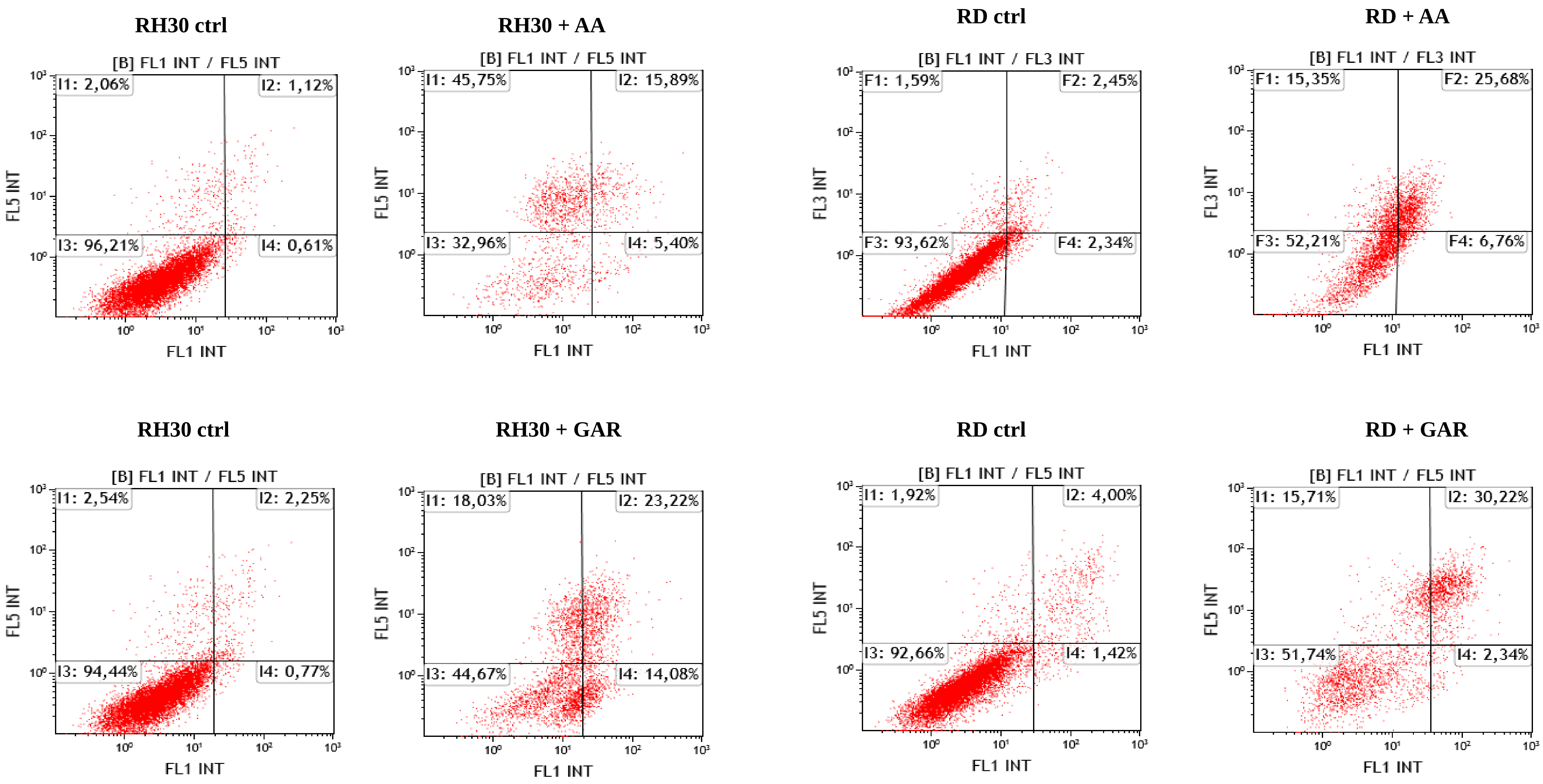

Supplementary Figure S3. Representative plots of the evaluation of the apoptosis of RMS cells under the influence of high doses of AA (55 µM) and GAR (25 µM). Percentages indicate number of cells in particular stages of apoptosis.
